# Integrated Primary Healthcare Opioid Tapering Interventions: A Mixed-Methods Study of Feasibility and Acceptability in Two General Practices in New South Wales, Australia

**DOI:** 10.5334/ijic.5426

**Published:** 2020-10-22

**Authors:** Ruth White, Chris Hayes, Allison W. Boyes, Christine L. Paul

**Affiliations:** 1 Hunter Integrated Pain Service, Hunter New England Health, Newcastle, New South Wales, AU; 2 School of Medicine and Public Health, University of Newcastle, NSW, AU; 3 Hunter Medical Research Institute, Newcastle, NSW, AU

**Keywords:** integrated-care, opioids, chronic-pain, case-study, primary healthcare, deprescribing

## Abstract

**Introduction:**

Integrated team-based primary healthcare is well positioned to support opioid tapering for patients experiencing chronic pain. This paper describes the development, implementation and acceptability of a primary healthcare opioid tapering intervention ‘Assess Inform Manage Monitor’ (AIMM) at two sites.

**Methods:**

AIMM involved GP advice; nurse monitoring and potential engagement with: community pharmacist; psychologist; dietitian and exercise physiologist. Individuals receiving 90 days or more of prescription opioids were eligible. Patient and provider surveys and qualitative interviews were completed.

**Results:**

Of 140 eligible patients, 37 attended during the study period and were invited to participate. Patient post-intervention surveys (n = 8) and interviews (n = 6) indicated the intervention was acceptable, although the perceived value of some of the integrated team was low. GP and practice nurse support was valued. Providers (n = 4) valued team integration. Low weaning readiness was a barrier to engagement by patients and providers.

**Key lessons and conclusions:**

The intervention, whilst conceptually acceptable, was not feasible in its current form. Future efforts to transition patients towards integrated care should retain the practice nurse and place more focus on understanding and reinforcing patients’ readiness to wean. Greater inter-professional collaboration may also be needed. Such refinements may advance the cause of opioid reduction in primary care.

## Highlights

**What is already known about the topic?**

- Barriers to achieving opioid reduction in primary care include: variation in clinical practice of GP; lack of understanding in the community about the lack of benefit and high risk of harm from opioid therapy for chronic non-cancer pain; providers may not be co-located making multidisciplinary team discussion difficult; time constraints to organising a whole person team-based approach.

**Key learnings**

This case identified four key lessons:

- Patients’ readiness to reduce opioids was low and providers preferred to only ‘lightly broach’ the subject of tapering.
- Patients were open to team-based, integrated care, but reported non-nurse sessions as being of moderate or limited helpfulness, and had low levels of adherence to the team care plan.
- A trained practice nurse acting as a key supportive facilitator was perceived by patients as a helpful resource.
- Practitioners valued a team-based approach. In addition, GPs desired greater collaboration with and support from specialist pain medicine physicians to reinforce messaging about opioid reduction.

## Background

Primary care plays a central role in responding to patients who experience chronic pain, including both primary and secondary musculoskeletal pain [1,2]. Providing comprehensive and co-ordinated care for people reporting significant emotional distress and, or functional disability is however not simple to achieve. In these time-limited settings, treatment is often focussed on the prescription of opioids [3,6,7]. However, long-term opioid medication is not effective for improving chronic pain or reducing functional disability and is associated with many harms [8,9,10,11].

Referral to tertiary care offers pathways for some patients to decrease reliance on opioid medication [3,4]. Further, delivery of interdisciplinary cohesive care in tertiary settings has been shown to simultaneously improve outcomes such as physical functioning, sleep and pain- coping strategies [5,14,15,16]. There has been little research outside tertiary clinic settings into integrated interventions specifically targeting opioid reduction coupled with non-pharmacological management designed to enhance emotional well-being and reduce functional disability [17,18,19].

In Australia, it has been recognised that integrated care strategies that are non-medication focused are needed at a primary care level to address the extensive healthcare and societal burden for people experiencing chronic pain [7]. Despite this recognition, Australian Medicare items offer limited scope in a primary care context for uninsured people experiencing chronic pain to receive the multiple active treatment components offered in tertiary pain clinics, and currently no options exist for group-based visits [9,10]. Therefore, there is a specific need to develop and test approaches to creating access to a primary-care multidisciplinary team [24]. To our knowledge there has been no previous attempt in primary care in Australia to study the use of the national universal health insurance scheme (Medicare) to approximate a team-based approach for people experiencing chronic pain and utilising long-term prescription opioids.

In Australia, general practitioners (GPs) are reimbursed by the Government for integrated management of patients with chronic pain alongside another chronic condition via two Medicare items, namely General Practice Management Plans (GPMPs) and Team Care Arrangements (TCAs) [11]. The GPMP components include: problems and needs statement, patient goals, provider treatments; patient actions and date to review goals. Current regulation requires that there must also be at least two providers such as psychologists, physiotherapists; pharmacists; occupational therapists; exercise physiologists; social workers and dietitians, aside from the GP involved in care to access a GPMP [12]. Finally, providers agree to engage in collaborative two-way communication regarding the GPMP and TCA. Further, Medicare also funds up to 10 individual psychological services under the ‘Better Access’ initiative.

Non-Australian data support the potential of primary-care-based multidisciplinary treatment to offer non-pharmacological alternative strategies to treat chronic pain. Globally, there are very few primary care based studies reporting on feasibility and acceptability of behavioural support for opioid reduction. Recently, in the USA, Vogler and colleagues [13] reported a completion rate of 14% (5/35) for a series of four group visit 90-minute interventions, with 74% of patient participants (26/35) reporting that they would recommend the intervention to others. In contrast, a Swedish group [14], reported a higher rate of completion 84% (59/70) for an intensive 90 hour group based intervention.

Recognising the challenge of implementation we set up and worked with a clinical reference group to develop an integrated intervention for primary care named the ‘Assess, Inform, Manage and Monitor’. Using a new community guideline designed to facilitate reduction in opioid dose [15], the integrated intervention was modelled on tertiary multidisciplinary team approaches, using an evidence-informed whole-person approach to assist people experiencing chronic pain and reliant on opioids to achieve opioid reduction by switching to non-medication behavioural alternatives. The theoretical behavioural framework used is the Behaviour Change Wheel (BCW) which incorporates the COM-B model [23]. The central hub of the BCW framework allows individual behaviour to be explained from three fundamental aspects: opportunity, capability and motivation. This means that for any behaviour to occur, the person has to have physical and psychological capability; have opportunity, both socially and physically; and be motivated. Around the hub are nine intervention functions: education, persuasion, incentivisation, coercion, training, restriction, environmental restructuring, modelling and enablement which aim to address deficits in one or more of the three conditions. Surrounding the intervention functions, an outer rim comprises seven policy options: communication/marketing, guidelines, fiscal measures, regulation, legislation, environmental/social planning and service provision which provide context and can be utilised to help deliver the intervention functions.

The AIMM model of care engages a geographically available and supportive multidisciplinary team who receive joint training in a pro-recovery, whole-person approach focusing on 5 key areas: biomedical, mindbody, connection, physical activity and nutrition [14,16,17,18]. Patients are ***assessed*** by their GP to ensure serious pathologies (red flags) are eliminated, their beliefs regarding ongoing pain and its meaning are explored and the messaging that pain is not a symptom of damage is reinforced. Patients are ***informed*** of current evidence and the rationale for tapering opioids in line with community guidelines [15,19,20]. The practice nurse role is to discuss relevant behavioural change ***management*** *options* including team-based planned care (via Medicare-subsidised visits with a dietitian, exercise physiologist, an accredited practicing pharmacist medication review and a psychologist when indicated) plus encourage and undertake regular ***monitoring*** [18].

## Problem Statement and Goal

Despite the promise of an integrated primary care approach, it was unknown whether this patient group would actively attend and complete such an intervention. Therefore, a pilot-study was undertaken to establish the feasibility and acceptability of an individually tailored combination of non-opioid treatment choices, from the perspective of patients and healthcare providers to assist in refining the model prior to an efficacy trial.

The goal of this paper is to describe the development and implementation of the multicomponent, integrated primary healthcare opioid tapering intervention ‘Assess Inform Manage Monitor’ (AIMM) in the context of two sites in NSW, Australia; evaluate the feasibility (i.e. treatment completion rate) and describe its acceptability (i.e. post session satisfaction) from both patient and provider perspectives; and determine key lessons for future intervention iterations.

## Methods

### Design

A mixed-method approach was used to evaluate patient and provider perspectives regarding the opioid tapering intervention Assess, Inform, Manage and Monitor.

### Setting

The research was carried out at two general practices in New South Wales, Australia. In terms of relative socio-economic disadvantage, both practices were ranked in the bottom 30% of Australian local government areas. Principal GPs at the two practices had an interest in opioid deprescribing and were estimated to be caring for more than 50 patients experiencing chronic pain (for the purposes of the GPMP/TCA this classifies as a chronic condition) and currently utilising long-term prescription opioid analgesics (for the purposes of accessing funding under GPMP/TCA, this becomes a second chronic condition).

### Description of AIMM Program

A schematic diagram of the AIMM schedule is provided as Table 1. This schedule represented the maximum self-management support available under current Medicare benefit scheduling. For example, as well as accessing regular medical and nursing support, eligible patients can access up to five psychologically informed accredited exercise physiologist or physiotherapist sessions or accredited practicing dietitian sessions. Further, patients can access a home medication review from a pharmacist and access individual psychology sessions. Eligibility screening and flagging of the medical record were followed by an invitation to participate. The behaviour change components of the intervention were then flexibly implemented as per the schedule. The final phase involved completion of a three-month review.

**Table 1:** Schematic diagram of timeline for the 12 weeks of a study participant receiving the maximum self-management support in aimm + review.

	Medical record screen	Invited to study	Week										

	2 weeks prior	1–2 week prior	1	2	3	4	5	6	7	8	9	10	12 weeks + 1 day after completion of GPMP/TCA

Eligibility screening occurs -including opioid dose from patient medical record. Record flagged	Participant attendance not required												
Patient invited, consented and completes initial AIMM baseline survey. (Survey received by researchers and summary forwarded to GP- prior to study week 1)		✓											
Intervention-Medical -AIMM survey assessment broader discussion^1^ -Complete planning phase GPMP/TCA^2^ -Regular monitor^3^ -Review GPMP/TCA^4^			✓^1^	✓^2^		✓^3^		✓^3^		✓^3^		✓^3^	✓^4^
Intervention-Nursing -Co-complete GPMP/TCA^2^ -Regular supportive care/monitor^3^ -Review GPMP/TCA^4^				✓^2^		✓^3^		✓^3^		✓^3^		✓^3^	✓^4^
Intervention- Psychology* If required up to 10 × sessions					✓	✓	✓	✓	✓	✓	✓	✓	
Intervention-Accredited Pharmacist Home Medication Review					✓								
Intervention-5 × Psychologically informed accredited exercise physiologist or physiotherapist sessions & accredited practicing dietitian sessions						✓	✓		✓		✓	✓	
Complete-AIMM 3/12 survey													✓

* If patient has elevated psychological distress at initial screening, this element of the intervention may be commenced *prior* to the remainder of the intervention (Up to 10 × Psychology sessions).

### Participants

#### Patients

Eligible patients were English speaking adults attending a follow-up consultation at the practice who were experiencing chronic pain and had accrued ≥90 days of prescription opioid medication use. We defined these patients as long-term opioid users. Patients with any of the following criteria were not enrolled: presence of red flags [19] indicating possible serious underlying pathology (such as bowel obstruction, perforated viscous, intra-abdominal sepsis, fracture, malignancy, cauda-equina syndrome, haemorrhage, thrombosis and meningitis); pregnant; in receipt of workers compensation benefits; had engaged a lawyer regarding pain status; awaiting a pain-related surgical procedure; receiving radiotherapy or chemotherapy for cancer and/or are receiving palliative treatment or care; living in an aged-care facility; physically or mentally unable to complete survey; current abuse of illicit substances; unable to use a telephone due to cognitive or hearing impairment or had plans to move or be away for 6 weeks or more during the study period.

#### Providers

Eligible providers were a group of healthcare professionals associated with the two general practices including: GPs, chronic disease practice nurses, accredited practicing dietitians, accredited exercise physiologists, accredited practicing pharmacists and clinical psychologists. Providers needed to be willing to provide their respective healthcare services, that is, the intervention components, using GPMPs and TCAs without charging additional fees. One practice nurse acted as an internal clinical facilitator at each site.

### Procedure

#### Patient recruitment and follow up

The electronic medical records of the study sites were interrogated by the practice manager to identify and generate a list of potentially eligible patients. These records were then screened by the practice GPs to identify any for whom there were safety concerns or contraindications as outlined for study participation. Those considered eligible to participate had their medical record electronically ‘flagged’ by the practice manager. When a patient with a flagged medical record attended the practice, the GP followed a prepared script to provide verbal information and invite participation in the AIMM pilot study. In order to optimise recruitment, a range of strategies were implemented including regular site visits to meet practice clinical staff and to follow-up potential patient participants (by RW); provision of a monthly recruitment progress report to study sites; re-imbursement gift cards to patient participants who completed the study; laminated ‘recruitment’ prompt cards for GPs and use of a research assistant to facilitate data collection at one of the study sites.

Interested patients were invited to sit with the practice nurse, who was trained to explain the study and provide a Participant Information Statement and Informed Consent Form. Two initial study appointments with the practice nurse were made for consenting patients; firstly, to complete the baseline electronic questionnaires and secondly to complete the GPMPs and TCAs guided by the summary results received from the researchers in the following week (including possible referral for Medicare funded (free) psychologist visits).

The practice nurse or a research assistant electronically collected the post intervention survey 3 months following baseline questionnaire completion.

#### Provider recruitment, training and follow up

The practice managers identified multidisciplinary providers linked to the practice and currently providing GPMP and TCA based chronic disease care. All providers received a two-step intervention training package. Step one was responding to a web link and completing an online pain attitude questionnaire plus spending 30 minutes reviewing a specialist pain website in the week prior to the next step. The second step was attendance at two face-to-face, two-hour interactive workshops led by pain experts. The workshops included: completion of a website engagement survey; discussion of the rationale and benefits of opioid reduction and switch to broader behavioural treatments plus in-depth consideration of clearly defined roles.

Implementation of AIMM required each health care provider to build confidence in their new roles as stewards of behaviour change in pain management. The intervention training package included sessions on understanding changing roles for each of the health care providers including GP; practice nurse; psychologist; accredited pharmacist; dietitian; physiotherapist or accredited exercise physiologist. Treatment was not rigidly directed, instead providers were given flexibility to tailor the behaviour change components to the individual patient.

Following exploration of roles using role plays, a post-workshop pain attitude questionnaire concluded the training. A mixed subset of health care providers was asked to participate in a 10-minute semi-structured telephone interview at completion of the intervention period. The training package has been described in detail elsewhere [20].

#### Evaluation Measures

The mixed-methods evaluation of the intervention involved completion of (1) a pre- and a post- intervention survey of patients and (2) a post-intervention semi-structured telephone interview with patients and their multidisciplinary healthcare providers (Appendix 1 & 2). Interviews were conducted by one author (RW). The length of the interview varied with degree of participant engagement. Patient participants received a $40 supermarket voucher as reimbursement for completing the telephone interview.

#### Pre-intervention patient survey

*Demographic characteristics* included gender, age, indigenous status, educational level, internet access, income source, employment and housing status.

*Clinical characteristics* included duration of pain experience; pain severity and interference (using the 7-item Brief Pain Inventory) [21]; previous exposure to ‘talking treatments’; prescribed opioid intake (using a visually-aided checklist of medication names, dosage strengths and number of daily doses); and readiness to wean off opioids with four options: ready in the next 30 days; ready in next 6 months; may be ready in the future or never expect to wean off.

#### Post-intervention patient survey

A 10-item study-specific survey was developed by the authors to assess acceptability of the key components of the intervention. Patients were asked to indicate:

*Helpfulness* of the support provided (6 items) by each of the following healthcare providers: GP regular review and support to wean off opioids, practice nurse supportive care, Home Medication Review with accredited pharmacist, accredited practicing dietitian sessions for planned dietary changes, accredited exercise physiologist sessions for planned physical activity component and additional psychologist consults for pain management psychology skills. Response categories ranged from 0 = completely unhelpful to 4 = completely helpful and 5 = not applicable.

*Satisfaction* with the healthcare sessions (3 items). The overall number of healthcare provider sessions; the different mix (types) of sessions and the duration of the sessions. Satisfaction scored from 0 = completely unsatisfied to 4 = completely satisfied.

*Global impression of change* (1 item) was based on the Patient Global Impression of Change scale and asked participants to indicate their impression of overall change with AIMM. Responses ranged from 0 = very much worse to 6 = very much improved [22].

#### Post-intervention patient interview

The authors developed a semi-structured telephone interview to explore patients’ experiences with each of the intervention components. Seven open-ended questions asked participants to reflect on how each component of the intervention and the overall experience influenced their understanding of and approach to living with the experience of ongoing pain. Concern about weaning off opioids was specifically prompted during the interview. Other aspects included travel to appointment, time involved and costs incurred (e.g. petrol, taxis). Interviews, were audio recorded with participant’s consent, and independently transcribed.

#### Post-intervention healthcare provider interview

Key informant interviews explored the providers’ views about the feasibility of implementing the multidisciplinary approach and particularly the opioid weaning component; as well as questions regarding acceptability, that is, what worked well and what could be changed or improved. Interviews were audio recorded with consent and transcribed.

## Data analysis

Descriptive statistics were used to summarise the survey data. Means and standard deviation, or frequency were calculated using Stata/IC 13.1. Interview transcripts were independently coded by one author (RW) and reviewed by another author (CH). The researchers reviewed and discussed discrepancies until agreement was reached. N-Vivo software was used for the coding. Procedures were informed by modified grounded theory utilising an iterative analysis process throughout the data collection period [23]. We applied descriptive phrases to each concept that emerged from both patient and healthcare provider participants.

## Ethical Considerations

This study received ethics approval from the Hunter New England Health and University of Newcastle Human Research Ethics Committees. HNEHREC reference No:15/10/21/5.01 NSW HREC Reference No: LNR/15/HNE/371 SSA Reference No: LNRSSA/15/HNE/372.

## Results

### Feasibility

Of the 140 patients identified as eligible to participate, 37 attended the practice during the trial period and were invited by the practice nurse to participate in the study. From these 37 patients, 18 attended a practice nurse consultation, representing an enrolment rate of 48%. All 18 patients completed the baseline survey, had a medication review and developed an individualised GPMP and TCA. Of these 18 patients, 8 completed follow-up acceptability questionnaires and 6 were able to be contacted and completed a telephone interview. 10 patients were lost to follow-up.

Participant characteristics are described in Table 2. More than half were women (n = 14); most were in receipt of a Government Pension or benefit (n = 17) and most had been experiencing pain for 5 years or more (n = 13). Readiness to wean off opioids was low with only one patient reporting being ready to wean in the next 30 days.

**Table 2:** Baseline characteristics of the patient study sample (n = 18).

	Mean (SD)

Average age (years)	52.77 (11.41)
Pain Intensity (measured on Brief Pain Inventory *)	5.90 (1.53)
Pain Interference (measured on Brief Pain Inventory*)	6.51 (2.14)
Average daily morphine equivalent (mg)	133.27 (154.61)
	**N**

Female	14
**Indigenous status**	
Aboriginal or Torres Strait Islander	2
**Highest level of education**	
Primary School	4
School Certificate	7
Higher School Certificate	1
TAFE certificate or Diploma	5
University or other Tertiary Qualification	1
**Access to internet**	
No	7
Yes	11
**Income source**	
Government Pension or benefit	17
**Employment status**	
Employed (full or part time)	0
Unemployed	11
Retired	3
Other	4
**Housing Status**	
Property Owner	7
Renting	9
Living with friends/family	1
Other	1
Experienced pain >5 years	13
Previously received talking treatments	13
**Readiness to wean**	
Ready in next 30 days	1
Ready in next 6 months	5
May be ready in the future	10
Never expect to wean off	2

Higher score (range 0–10) represents higher level of pain intensity and interference with functioning [21].

### Acceptability of AIMM: patient perspectives

Patients’ perceptions regarding the helpfulness of the support provided by each member of the multidisciplinary team are displayed in Table 3. Half of the respondents reported that working with the practice nurse was a helpful component of the intervention. Despite all patients developing a GPMP, most reported the support provided by the pharmacist, exercise physiologist, dietitian and psychologist as either unhelpful or not applicable.

**Table 3:** Acceptability of healthcare provider support n = 8.

	n					

	Completely unhelpful	Unhelpful	Neither unhelpful or helpful	Helpful	Completely helpful	Not applicable

Attending general practitioner for regular review and support sessions to improve confidence and motivation to wean off long term opioid therapy and understand pain	1		2	3		2
Working with the practice nurse to develop a management plan and attending regular support sessions to improve confidence and motivation to self-manage pain		1	2	2	2	1
Having a home pharmacist visit to improve confidence and motivation to wean off opioids	1	1	1	1	1	3
Attending the dietitian sessions to improve confidence and motivation to make planned dietary changes	2	1	1	2		2
Attending the exercise sessions to improve confidence and motivation to make planned physical activity changes	1	1	1	1	1	3
Additional psychologist support to improve capability and confidence in applying psychological skills	1		1	1	1	4

In terms of satisfaction, most patients (n = 5) reported being either satisfied or completely satisfied with the number and mix of sessions offered. The remaining three patients were neither satisfied, nor unsatisfied. Half the patients reported positive global change, 3 patients reported no change and 1 reported being somewhat worse.

Qualitative analysis of patient interviews identified two major salient themes.

The first theme was labelled ‘lack of readiness for opioid reduction and problems with weaning’. Patients used strong language to express why they had not attempted weaning during the intervention and expressed fear surrounding past weaning attempts:

‘Yeah. I can hardly move, and then when I start taking it again because we tried weaning it before … I couldn’t move for three days. I was in bed. I could not move because of the pain’ (Female age 43)

‘No way because I know on a day where I, because I tried that a few months ago, and on just one day of missing out I just couldn’t get out of bed. Sorry, yeah I’m still on the same medication, the pain, not much better, I’ve still got the pain and everything’ (Male age 59)

Only one patient was ‘ready to wean’ and she stated during her interview that she was ‘focused’ and had a reason or goal for why she wanted to taper the opioids to cessation.

‘Yeah, so I just had that determination in me so, because I’ve got a cruise in February, so I’m like, right, I’ve got a cruise and I’m sick of being on this medication, so I was just like, bang’ (Female age 39)

Another patient who tapered slightly stated she

‘Came down a bump because she did not want to become addicted to them’ (Female age 55)

The second theme to emerge surrounded the support being offered by the healthcare providers and was labelled ‘supportive contact’.

Most of the interviewees spoke favourably of their regular encounters with the practice nurse.

‘Yeah, extra bit of support and plus knowledge too of different medications, and then getting me into, like I said, the dietitian and different people in the organization just to sort of help me get to relieve this pain, make this pain easier to deal with’ (Female age 43)

‘The nurse that did it, was really good talking to me about coming down off the morphine, and the other meds” (Female age 57)

Referrals for non-medication behavioural treatments were also found to be acceptable for short term treatment intervention when the patient could see the value in the referral.

‘yeah, they’ve actually given me a referral to see their dietitian and because my arthritis is … I’ve put on a little bit of weight so it’s hurt my knees’ (Female age 47)

‘I’ve seen a dietitian and all that, and the exercise place, I went and joined the gym and all that…and just do light exercises’ (Male age 59)

However, some patients expressed negative experiences in relation to the support provided:

‘I saw her twice, then I actually stopped going because she (the psychologist) wasn’t dealing with any of the different things I could do for the pain’ (Female age 53)

And

‘I think it would be better if the appointments were closer together…it’d be good if more things could happen’ (Female age 39)

### Healthcare providers

Nineteen multidisciplinary healthcare providers participated in the pilot study. This included GPs (n = 7, of which 5 were male); practice nurses (n = 5, of which 4 were female); exercise physiologists (n = 2 both male), dietitian (n = 1, female), community pharmacists (n = 2, 1 male) and psychologists (n = 2, both female). Of these, four providers including GP (n = 1), practice nurse (n = 1), dietitian (n = 1), pharmacist (n = 1) agreed to a telephone interview.

### Acceptability of AIMM: provider perspectives

Two themes emerged from the provider interviews. The first theme was labelled ‘collaborative care’ and explored providers’ views of the feasibility of being involved in a collaborative primary care based team:

‘I think that any collaboration between the different healthcare professionals is always going to benefit the patient’ (Pharmacist, female)

‘Well, I think it makes sense, seeing as though the whole idea of a General Practice Management Plan is to address chronic disease. And certainly, you know, pain plays a major part in a lot of people’s, you know, health and wellbeing, so, yeah, I think it makes sense’. (Dietitian, female)

We also asked providers whether they had any other comments about any aspect of the pilot study. One GP indicated that future iterations of integrated care should include enhanced primary-tertiary team linkage:

‘I think the concept of devolving pain management out into primary care probably means that there need to be maybe stronger, more regular interactions between all the people involved’ (GP, male)

The second theme was labelled ‘opioid weaning concerns and maintaining the status quo’. Providers were in agreement that providing primary healthcare team-based pain management was acceptable to them, however patient compliance was challenging. Clinicians noted that few patients were actually ready to take action to wean their prescribed opioids. One practice nurse noted that whilst patients may have considered alternatives they remained ‘petrified’ of the weaning process as they felt that only another medication could be substituted for the reduced opioid. Examples of quotes include:

‘I think they were just too scared of what would happen to their pain management without the drugs’ (GP, male)

‘Well, I think they’re probably naturally sometimes a harder group to motivate from step one but yeah, certainly I would say as a whole, they’re probably quite hard to motivate’ (Dietitian, female).

‘I like just too lightly broach the subject…I don’t like to get people off side too much if I can help it …I mean to be perfectly honest of all the people I saw I had one person who was really intent on getting off opioids and really wanted to do it and, I think, did…..Looking at alternatives, I think maybe in some cases initially they went, “Oh, yeah, that sounds all right ..and maybe thought about it for a couple of days and went, “Oh my God, no”. That was probably a difficulty… people just went, “Oh, no, I don’t think I can do that”’ (Practice Nurse, male).

## Discussion and lessons learned from the integrated care case

This study focused on patient participants who were accessing their primary care physician to receive prescription opioids to manage chronic pain; and their primary care providers. The current work was the pilot phase of a primary-care-based multidisciplinary intervention targeting long-term opioid use and aiming to taper use and transition to non-pharmacological modalities of care. We developed a fuller understanding from patient and provider views, of the usefulness of the different intervention components and suggestions from both patients and healthcare providers working in primary care of how to better address this complex problem.

We planned originally for this pilot study to involve recruitment of approximately 100patients enrolled from two different primary care practice settings, following the same procedures. We were unsuccessful in recruiting the desired number of patient participants, uncovering possible attitudinal barriers and adherence issues. Patient drop-out was another major concern. It must be acknowledged that while the low patient recruitment rates by GPs to AIMM may be partly due to a reluctance among patients to reduce opioid use, it is possible that reasons for reluctance to engage with AIMM were more complex. Possibilities include a reluctance to engage with research, or a reluctance of GPs to change practice, both options are possible though remain speculative and represent a degree of complexity which this study was not designed to clarify.

Analysis of the interviews revealed a number of potential themes on which future iterations of the intervention could focus to improve uptake.

### Attitudinal Barriers

Firstly, we identified ‘lack of readiness’ to wean amongst those patients who did enrol in the study. The patients in our study mostly reported their past experiences with tapering as ‘not going well’. Patients expressed fear of the impact of reduced opioids and this was recognised by their GPs. The nurses on the team were particularly conscious of remaining ‘on-side’ with the patient during tapering conversations. Given this status quo it is perhaps not surprising that weaning did not readily occur. Our protocol did not clarify specific tapering goals or offer specialist support for GPs to proceed with opioid reduction in the face of patient reluctance and this could be viewed as failure at the ‘intervention function’ level to ensure effective tapering.

The second theme to emerge involved therapeutic support and opportunity to access integrated care. While patients and providers like the idea of team care, there were significant barriers to patients actually using it. In our study, practice nurses were integral for supporting patients and linking them with broader supportive care. This role for nurses as care co-ordinators has been reviewed, though to date there is no direct evidence that clinical outcomes are impacted by such a role [24]. Providers considered integrated care as helpful, however this case demonstrated that many patients placed less value on switching to alternative approaches as a long-term pain self – management strategy compared with the status quo of remaining on prescription opioids.

An important strength of the study was the use of a strong theoretical framework to guide the development of our complex intervention for people experiencing chronic primary pain [25]. Yet, despite targeting all three central components of behaviour change according to the BCW, that is, opportunity, capability and motivation, we found it difficult to engage patients in the intervention.

Despite uptake of the intervention being low, we were able to test the feasibility and acceptability of the AIMM approach across two practices operating in a low socioeconomic environment with access to a wider health team and gather valuable feedback for future iterations of the intervention.

Given that these findings suggest the intervention requires refinement, future iterations of the intervention would benefit from both better support for patients and providers around their readiness or motivation to wean as well as better preparation of the patients and multidisciplinary team such that they are fully integrated and working together, possibly including a stronger connection with the tertiary pain service for the providers.

Specifically, from the provider perspective, the results from this study would suggest a stronger focus on the GP enacting a ‘boundary holding’ or ‘restriction intervention’ and engaging patients in regular preparatory tapering conversations. This BCW perspective allows the patients’ reflective motivation (beliefs about consequences) to slowly be modified as they come to believe that opioids are harmful in the long-term.

From the patient perspective, fears of future functional decline and loss of hope regarding functional recovery would be considered by a core GP and practice nurse pain team as a malleable concept.

Other pain team members would be utilised when clinically relevant e.g. in the presence of significant depression, trauma or anxiety. For the most part, practice nurse involvement regularly allaying fears regarding the impact of opioid reduction on peoples’ daily lives will be an important aspect to incorporate as the central ongoing support.

## Conclusion

AIMM was the first iteration of an integrated approach to implementing whole-person care for people experiencing chronic pain, intended to reduce reliance on long-term prescription opioids and transition to non-pharmacologic treatment modalities. Several aspects of the intervention were not implemented as planned. Patients’ level of readiness to wean is important. An engaged and supportive practice nurse is one element that facilitates a range of healthcare providers to engage. Providers value team-based care and desire greater inter-professional links with their colleagues. It is possible that greater use of prescribing boundaries by GPs and stronger opioid policy direction by regulators may facilitate future approaches to opioid weaning.

Although only a first step, these preliminary results may assist in developing a future more effective primary-care-based opioid tapering intervention.

## Additional Files

The additional files for this article can be found as follows:

10.5334/ijic.5426.s1 Appendix 1. Telephone script-patient – feasibility/acceptability of AIMM V-27/10/15.

10.5334/ijic.5426.s2 Appendix 2. Telephone script-health care providers V-22/02/15.
