# Supplementary material for: Integrated Primary Healthcare Opioid Tapering Interventions: A Mixed-Methods Study of Feasibility and Acceptability in Two General Practices in New South Wales, Australia
Source: Int J Integr Care. 2020 Oct 22;20(4):6. doi: 10.5334/ijic.5426 (PMC7583216; doi:10.5334/ijic.5426)
Supplement: Appendix 1. — Telephone script-patient – feasibility/acceptability of AIMM V-27/10/15. [file ijic-20-4-5426-s1.pdf]

Appendix 1

Telephone Script-Patient- Feasibility/Acceptability of AIMM V-27/10/15

Telephone interview schedule for patients who have completed the AIMM pilot study

Date: _____________________ Participant No:

Good morning/good afternoon, my name is _________. You were invited to take part in the research project being jointly conducted by Hunter New England Local Health District and University of Newcastle. Firstly, we would like to thank you for participating in the:

Assess, Inform, Manage and Monitor (AIMM) pilot study for people with chronic pain being managed with chronic opioid therapy in an Australian primary care setting.

Is now a good time to discuss *your honest and open thoughts* about the study further over the phone?

It is expected the interview will take around 15 minutes to answer 7 questions and you will be asked mid-way if you wish to continue

1. To begin, can you tell me whether you were offered any or all of the following health provider appointments? (psychology, home medication review, dietitian, exercise physiologist or physiotherapist, practice nurse- who would have helped with the management plan, general practitioner appointments). *Ask each in turn.* [Enquire as to] how long the person had to wait (days? Weeks?) for ***health professional appointments***? (Need to identify whether the option of having the appointments was raised and if so, did patient accept or decline)

____

____

____

2. Thinking about ***how far you had to travel*** (time and costs e.g. petrol involved) to attend the appointments. Do you think this affected your ability to attend your appointments?

____

____

____

3. Were the ***appointment times*** convenient? Did this affect your ability to attend your appointments?____

____

____

4. Were the appointment times ***long enough*** with the health professionals? (How supported did the person feel with the health professionals?)____

____

____

You have now completed 4 of the 7 questions, there are three questions remaining, do you wish to continue now?

If no, reschedule

If yes, proceed

5. In your opinion, could you now tell me about which aspects of the AIMM approach ***worked well***? Follow with, we are particularly interested in whether this was your first attempt at weaning off opioids? Or second? Or third? etc. and what it was that helped you decide to wean now? i.e., was there any particular reason- such as the offer of support through the study, a health scare? A life event that happened? Something coming up- e.g. a holiday? or no particular reason? - that meant you were ready?

____

____

____

6. In your opinion, could you now tell me about which aspects of the AIMM ***approach did not work well?*** (I am particularly interested in whether you *travelled to different places* to work with health professionals or not-and how that experience was for you/ any aspects of the weaning process that did not work well?)

____

____

____

____

7. If you have any other questions or comments about any aspect of the pilot study, negative or positive, I would appreciate your views. What is next for you (continue weaning/ other?)____

____

____

____

____

____

____

To conclude…

On behalf of the researchers, the University of Newcastle and Hunter New England Local Health District we thank you for your time today and again for having participated in the research

Goodbye
