# Supplementary material for: Integrated Primary Healthcare Opioid Tapering Interventions: A Mixed-Methods Study of Feasibility and Acceptability in Two General Practices in New South Wales, Australia
Source: Int J Integr Care. 2020 Oct 22;20(4):6. doi: 10.5334/ijic.5426 (PMC7583216; doi:10.5334/ijic.5426)
Supplement: Appendix 2. — Telephone script-health care providers V-22/02/15. [file ijic-20-4-5426-s2.pdf]

APPENDIX 2

Telephone Script-Health Care Providers V- 22/02/15

***Telephone interview schedule for healthcare providers who have participated in the AIMM pilot study***

Date: ______________________ Participant No:________________

Good morning/good afternoon, my name is _________. You were invited to take part in the research project being jointly conducted by Hunter New England Local Health District and University of Newcastle. Firstly, we would like to thank you for participating in the:

*Assess, Inform, Manage and Monitor (AIMM) pilot study for people with chronic pain being managed with chronic opioid therapy in an Australian primary care setting.*

Is now a good time to discuss *your honest and open thoughts* about the study further over the phone?

It is expected the interview will take around 30 minutes to answer all 6 questions and you will be asked midway if you wish to continue

1. This question asks you *about the feasibility of routinely using a chronic pain General Practice Management Plans and Team Care Arrangement* framework. In your opinion, could you tell me how the organisational support to embed patient information into the template has impacted on your practice? (*Ask whether the resources, which were developed for time poor clinicians, were easy to use/ was there too much or too little information?*___________________________________________________________________________

   ___________________________________________________________________________

   ___________________________________________________________________________

   ___________________________________________________________________________

   ___________________________________________________________

2. Could you tell me about how well the AIMM multidisciplinary appointment schedule worked for you? Do you think it is *feasible* to be part of an opioid reduction focused multidisciplinary pain team providing regular/consistent key message to the patients? *(collaborative nature of pain team)*

   ___________________________________________________________________________

   ___________________________________________________________________________

   ___________________________________________________________________________

____________________________________________________________________________________________________________

__________________________________________________________________________________

3. Can you tell me now how *acceptable*/how satisfied you found the training you were given i.e. 30 minutes session familiarising yourself with the pain resources available on Hunter Integrated Pain Service website and a ½ day active learning workshop plus follow up mentorship over 12 weeks (*how interesting was the training? How satisfied were you with the quality of the training and mentorship)?*

____________________________________________________________________________________________________________

____________________________________________________________________________________________________________

____________________________________________________________________________________________________________

____________________________________________________________________________________________________________

____________________________________________________________________________________________________________

____________________________________________________________________________________________________________

______________

*You have now completed 1/2 of the interview, there are a further 3 questions, do you wish to continue now?*

*If no, reschedule*

*If yes, proceed*

4. Thinking about the time you had to learn the key messages and 4 communication habits-how confident you were (or now are) to deliver the intervention-how *acceptable was learning* (pre disposing activity/ ½ day workshop/reading and 3/12 mentorship*/5 key messages of complex system-whole person model/4 communication habits/*)

____________________________________________________________________________________________________________

____________________________________________________________________________________________________________

____________________________________________________________________________________________________________

____________________________________________________________________________________________________________

____________________________________________________________________________________________________________

_______________________________________________

5. In your opinion, could you now tell me about which aspects of the AIMM approach worked well–were *acceptable*/not so acceptable? (attitudes about stewardship for pain medicine/ knowledge of professional role and confidence in managing and monitoring patients as part of an opioid reduction team/ *were patients easy to activate? /concerns/barriers? / did patients say they did not like any aspect?)*

____________________________________________________________________________________________________

____________________________________________________________________________________________________

____________________________________________________________________________________________________

____________________________________________________________________________________________________

_____________________________________________________________________________

6. If you have any other questions or comments about any aspect of the pilot study, negative or positive, I would appreciate your views *(Is provider still using any of the resources/any of the skills? -If so, which parts are still be used?*

____________________________________________________________________________________________________

____________________________________________________________________________________________________

____________________________________________________________________________________________________

____________________________________________________________________________________________________

_____________________________________________________________________________

*To conclude…*On behalf of the researchers, the University of Newcastle and Hunter New England Local Health District we thank you for your time today and again for having participated in the research. Goodbye.
